# Ignoring non‐English‐language studies may bias ecological meta‐analyses

**DOI:** 10.1002/ece3.6368

**Published:** 2020-05-29

**Authors:** Ko Konno, Munemitsu Akasaka, Chieko Koshida, Naoki Katayama, Noriyuki Osada, Rebecca Spake, Tatsuya Amano

**Affiliations:** ^1^ School of Natural Sciences Bangor University Gwynedd UK; ^2^ Institute of Agriculture Tokyo University of Agriculture and Technology Fuchu Japan; ^3^ Institute of Global Innovation Research Tokyo University of Agriculture and Technology Fuchu Japan; ^4^ Plant Chemical Ecology Technische Universität Darmstadt Darmstadt Germany; ^5^ Biodiversity Division Institute for Agro‐Environmental Sciences NARO Tsukuba‐shi Japan; ^6^ Laboratory of Plant Conservation Science, Faculty of Agriculture Meijo University Nagoya Japan; ^7^ School of Geography and Environmental Science University of Southampton Southampton UK; ^8^ School of Biological Sciences The University of Queensland Brisbane Qld Australia; ^9^ Centre for Biodiversity and Conservation Science The University of Queensland Brisbane Qld Australia

**Keywords:** language barriers, publication bias, reporting biases, risk of bias, systematic review, obstacles linguistiques, biais de publication, biais de notification, risque de biais, revue systématique

## Abstract

Meta‐analysis plays a crucial role in syntheses of quantitative evidence in ecology and biodiversity conservation. The reliability of estimates in meta‐analyses strongly depends on unbiased sampling of primary studies. Although earlier studies have explored potential biases in ecological meta‐analyses, biases in reported statistical results and associated study characteristics published in different languages have never been tested in environmental sciences. We address this knowledge gap by systematically searching published meta‐analyses and comparing effect‐size estimates between English‐ and Japanese‐language studies included in existing meta‐analyses. Of the 40 published ecological meta‐analysis articles authored by those affiliated to Japanese institutions, we find that three meta‐analysis articles searched for studies in the two languages and involved sufficient numbers of English‐ and Japanese‐language studies, resulting in four eligible meta‐analyses (i.e., four meta‐analyses conducted in the three meta‐analysis articles). In two of the four, effect sizes differ significantly between the English‐ and Japanese‐language studies included in the meta‐analyses, causing considerable changes in overall mean effect sizes and even their direction when Japanese‐language studies are excluded. The observed differences in effect sizes are likely attributable to systematic differences in reported statistical results and associated study characteristics, particularly taxa and ecosystems, between English‐ and Japanese‐language studies. Despite being based on a small sample size, our findings suggest that ignoring non‐English‐language studies may bias outcomes of ecological meta‐analyses, due to systematic differences in study characteristics and effect‐size estimates between English‐ and non‐English languages. We provide a list of actions that meta‐analysts could take in the future to reduce the risk of language bias.

## Graphical abstract

Ignoring non‐English‐language literature may bias outcomes of ecological meta‐analyses, due to systematic differences in effect sizes between studies published in English and non‐English languages.

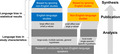

## 摘要

元分析在生态学和生物多样性保护定量证据的合成中起着至关重要的作用。元分析中估计的可靠性在很大程度上取决于对初级研究的公正抽样。虽然早期的研究已经探索了生态元分析的潜在偏差，但以不同语言发表的报告的统计结果和相关研究特征的偏差从未在环境科学中测试过。我们通过系统地搜索已发布的元分析，并比较现有元分析中包含的英语和日语研究之间的效应量估计值，来解决这种知识差距。在由日本研究机构下属机构撰写的40篇已发表的生态元分析文章中，我们发现有三篇元分析文章搜索了这两种语言的研究，涉及足够数量的英语和日语研究，产生了4个合资格的元分析（即三篇元分析文章中进行的四个元分析）。在这四个元分析的两个中，元分析中所包含的英语和日语研究之间的效应量差别很大，在排除日语研究时，导致总体平均效应量甚至其走向发生显著变化。观察到的效应量差异可能归因于英语和日语研究之间报告的统计结果以及相关研究特征，特别是分类和生态系统的系统性差异。尽管基于较小的样本量，我们的发现表明，由于英语和非英语语言之间在研究特征和效应量估计方面存在着系统性差异，因而忽视非英语研究可能会使生态元分析的结果有偏差。我们提供了一份元分析人员将来可能采取的一些行动的列表，以降低语言偏差的风险。

## Résumé

La méta‐analyse joue un rôle essentiel dans les synthèses de preuves quantitatives en écologie et en conservation de la biodiversité. La fiabilité des estimations dans les méta‐analyses dépend fortement d’un échantillonnage non biaisé des études primaires. Bien que des études antérieures aient examiné les biais potentiels dans les méta‐analyses écologiques, les biais n’ont jamais été mis à l’épreuve dans les sciences de l’environnement lors de publications dans différentes langues de résultats statistiques enregistrés et de caractéristiques des études associées. Nous abordons cette lacune dans les connaissances en recherchant de manière systématique des méta‐analyses publiées et en comparant les estimations d’ampleur de l’effet entre des études en anglais et en japonais figurant dans les méta‐analyses existantes. Sur 40 articles publiés de méta‐analyse écologique dont les auteurs sont affiliés à des institutions japonaises, nous trouvons que trois articles de méta‐analyse ont recherché des études dans les deux langues et ont mis en jeu des nombres suffisants d’études en anglais et en japonais, avec pour résultat quatre méta‐analyses admissibles (c.‐à‐d. quatre méta‐analyses effectuées dans les trois articles de méta‐analyse). Dans deux de ces quatre cas, les ampleurs de l’effet diffèrent de façon significative entre les études en japonais et en anglais comprises dans les méta‐analyses, ce qui provoque des changements considérables dans les ampleurs moyennes globales de l’effet et même dans leur direction lorsque les études en japonais sont exclues. On peut probablement attribuer les différences observées dans les ampleurs de l’effet entre les études en japonais et en anglais, en particulier en ce qui concerne les taxons et les écosystèmes, aux différences systématiques dans les résultats statistiques enregistrés, de même que dans les caractéristiques des études associées. Bien que basés sur un échantillon de petite taille, nos résultats suggèrent que le fait d’ignorer les études non anglophones peut biaiser les résultats de méta‐analyses écologiques en raison des différences systématiques dans les caractéristiques d’étude et les estimations d’ampleur de l’effet entre langue anglaise et langues non anglophones. Nous proposons une liste de mesures qui pourraient être adoptées à l’avenir dans les méta‐analyses pour réduire le risque de biais linguistique.

## 要旨

メタ分析は、生態学や生物多様性保全における定量的エビデンスの統合において重要な役割を果たしている。メタ分析における推定値の信頼性は、偏りのない一次研究の収集に大きく依存する。生態学におけるメタ分析で生じる様々なバイアスの可能性はこれまでの研究でも検討されてきたが、異なる言語で発表される統計結果や研究の特性にバイアスが存在するかどうかは、環境科学の分野では検証されたことがなかった。そこで本研究では、既存のメタ分析を系統的に検索し、そこで利用されている英語で発表された研究と日本語で発表された研究の間で効果サイズの推定値を比較した。日本の研究機関に所属する研究者が執筆した40の生態学におけるメタ分析論文のうち、3つのメタ分析論文のみが一次研究を2言語で検索し、その結果十分な数の英語研究と日本語研究を解析の対象としていた。そこで本研究では、この3つのメタ分析論文に含まれた4つのメタ分析を対象として用いた。4つのうち2つのメタ分析では、利用された英語研究と日本語研究で効果サイズが大きく異なり、日本語研究を除外した場合には、全体の平均効果サイズやその方向性までもが大きく変化することが明らかになった。このような効果サイズの違いは、英語研究と日本語研究で報告された統計結果や研究の特性（特に分類や生態系）が系統的に異なることに起因している可能性が高い。本研究では多くのメタ分析を対象にすることはできなかったものの、ここで得られた知見は、異なる言語で発表された研究間でその特性や効果サイズの推定値に系統的な違いがあるため、英語以外の研究を除外すると生態学におけるメタ分析の結果にバイアスが生じる可能性があることを示唆している。最後に、このようなメタ分析における言語バイアスのリスクを軽減するために有効であると考えられる対策についても提案を行う。

## Resumo

A metanálise desempenha um papel crucial na síntese de evidências quantitativas na ecologia e conservação da biodiversidade. A confiabilidade das estimativas nas metanálises depende fortemente da amostragem imparcial de estudos primários. Embora estudos anteriores tenham explorado possíveis vieses em metanálises ecológicas, os vieses nos resultados estatísticos relatados e características de estudos associados publicados em diferentes idiomas nunca foram testados em ciências ambientais. Abordamos essa lacuna de conhecimento pesquisando sistematicamente metanálises publicadas e comparando estimativas de tamanho de efeito entre os estudos em inglês e japonês incluídos nas metanálises existentes. Dos 40 artigos de metanálise ecológica publicados por autores filiados a instituições japonesas, descobrimos que três artigos de metanálise pesquisaram estudos nos dois idiomas e envolveram um número suficiente de estudos em inglês e japonês, resultando em quatro metanálises elegíveis (ou seja, quatro metanálises realizadas nos três artigos de metanálise). Em duas das quatro metanálises, os tamanhos de efeito diferem significativamente entre os estudos em inglês e japonês incluídos nas metanálises, causando mudanças consideráveis nos tamanhos de efeito médios em geral e até mesmo na sua direção quando os estudos em japonês são excluídos. As diferenças observadas nos tamanhos de efeito provavelmente são atribuíveis a diferenças sistemáticas nos resultados estatísticos relatados, bem como às características de estudos associados, particularmente táxons e ecossistemas, entre estudos em inglês e japonês. Embora baseados em um pequeno tamanho amostral, nossos resultados sugerem que ignorar estudos que não sejam em inglês pode influenciar os resultados de metanálises ecológicas, devido a diferenças sistemáticas nas características dos estudos e estimativas de tamanho de efeito entre o idioma inglês e o não‐inglês. Fornecemos uma lista de medidas que metanalistas podem adotar no futuro para reduzir o risco de viés de idioma.

## Resumen

El meta‐análisis juega un papel crucial en la síntesis de evidencia cuantitativa en ecología y conservación de la biodiversidad. La fiabilidad de las estimaciones en los meta‐análisis depende en gran medida del muestreo imparcial de los estudios primarios. A pesar de que estudios previos han explorado posibles sesgos en meta‐análisis ecológicos, sesgos en resultados estadísticos y características asociadas al estudio publicados en diferentes idiomas nunca han sido comprobados en ciencias ambientales. Abordamos esta brecha de conocimiento buscando sistemáticamente los meta‐análisis publicados y comparando las estimaciones del tamaño del efecto entre los estudios en inglés y japonés incluidos en los meta‐análisis existentes. De los 40 artículos de meta‐análisis ecológicos publicados por aquellos afiliados a instituciones japonesas, encontramos que tres artículos de meta‐análisis buscaron estudios en dos idiomas e involucraron un número suficiente de estudios en inglés y japonés, lo que resultó en cuatro meta‐análisis elegibles (i.e., cuatro meta‐análisis realizados en tres artículos de meta‐análisis). En dos de los cuatro, los tamaños de los efectos difieren significativamente entre los estudios en inglés y japonés incluidos en los meta‐análisis, lo que provoca cambios considerables en los tamaños de efectos medios generales e incluso su dirección cuando se excluyen los estudios en japonés. Las diferencias observadas en los tamaños de los efectos son probablemente atribuibles a las diferencias sistemáticas en los resultados estadísticos informados, así como a las características de los estudios asociados, particularmente los taxones y los ecosistemas, entre los estudios en inglés y japonés. A pesar de estar basados ​​en un tamaño de muestra pequeño, nuestros hallazgos sugieren que ignorar los estudios que no están en inglés puede sesgar los resultados de los meta‐análisis ecológicos, debido a las diferencias sistemáticas en las características del estudio y a las estimaciones del tamaño del efecto entre el idioma inglés y el no inglés. Proporcionamos una lista de acciones que los meta‐analistas podrían tomar en el futuro para reducir el riesgo de sesgo lingüístico.

## 1 INTRODUCTION

Global environmental change threatens ecosystems and biodiversity around the world (Ceballos et al., 2015; Díaz et al., 2019; WWF, 2018). A sound understanding of ecosystem responses to environmental drivers and human activities is therefore urgently required to inform policy and practice to mitigate against adverse ecological change (Sutherland, Pullin, Dolman, & Knight, 2004). It is increasingly demanded that this understanding draws on rigorous scientific evidence bases, best formed through the unbiased and systematic collation, appraisal, and meta‐analysis of primary empirical research (Pullin, 2012; Sutherland et al., 2004). Meta‐analysis can provide a powerful set of tools for summarizing the results of multiple studies, quantifying the variation in results among studies, and evaluating whether hypotheses are supported by the assemblage of existing studies (Gurevitch, Koricheva, Nakagawa, & Stewart, 2018; Koricheva, Gurevitch, & Mengersen, 2013).

A common criticism of meta‐analysis is the ignorance of potential biases during the search and selection of studies to be quantitatively synthesized (Borenstein, Hedges, Higgins, & Rothstein, 2009). Indeed, for a meta‐analysis to obtain a robust estimate of an overall true effect size, a random subset of all relevant primary studies should be included in the analysis. For example, because the nature and direction of a study's results can affect its likelihood of publication (publication bias: Bayliss & Beyer, 2015; CEE, 2018; Higgins & Green, 2011), the omission of unpublished data and gray literature may result in a biased sample of primary studies that give rise to an overestimated overall effect size (McAuley, Pham, Tugwell, & Moher, 2000; Turner, Matthews, Linardatos, Tell, & Rosenthal, 2008). Publication bias and its consequences for meta‐analysis are widely recognized, and mitigation measures to minimize this bias exist (Bayliss & Beyer, 2015; CEE, 2018). A much more overlooked bias in evidence synthesis is language bias, wherein the nature and direction of a study's results can affect the chosen language of its publication (Egger et al., 1997; Grégoire, Derderian, & Le Lorier, 1995; Higgins & Green, 2011; Juni et al., 2002). Omitting studies published in languages other than English, a common practice in meta‐analysis, could therefore also lead to a biased sample of primary studies. However, the prevalence and importance of language bias in ecological meta‐analyses have never been assessed to date (Livoreil et al., 2017). This is concerning, given that conclusions derived from biased meta‐analyses could lead to wasted resources if management actions are ineffectively prescribed or, worse still, may lead to unexpected or even perverse outcomes.

**Figure 1:**
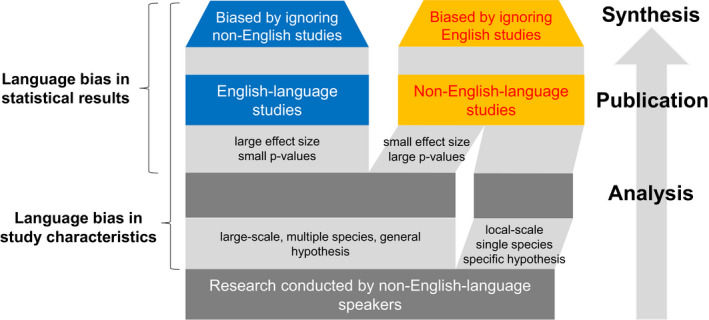
The fate of ignoring studies published in relevant language(s). Studies providing certain information (e.g., local‐scale studies focusing on specific hypotheses on a single species) may be more likely to be published in non‐English languages (*language bias in study characteristics*) because, for example, those studies tend to be conducted by local practitioners or they are often not of great interest from an international perspective. After the analysis, statistically significant or positive results may be more likely to be published in higher‐impact, English‐language journals (*language bias in statistical results*)

Previous studies of language bias in medical science have revealed differences in statistical results between publication languages (Egger et al., 1997; Grégoire et al., 1995; Juni et al., 2002). Referred to as “English‐language bias” (Egger et al., 1997) or “Tower of Babel” bias (Grégoire et al., 1995), it has been shown that positive or statistically significant results are more likely to be published in English than other languages (*language bias in statistical results* in Figure 1). This focus on statistical results is presumably because medical meta‐analysts are typically concerned with estimating the overall effects of treatments on a single species (i.e., *Homo sapiens*) under controlled conditions (e.g., the effectiveness of a drug at reducing symptoms of a disease). In contrast, ecological meta‐analyses are typically interested in variation among effect sizes and attributing this variation to meaningful covariates that vary among studies, such as species biogeographical contexts and intervention intensity (i.e., effect modification). Ecological meta‐analyses thus typically combine heterogeneous studies on a wide range of organisms and ecosystems (Gurevitch et al., 2018; Koricheva et al., 2013). Doing so may give rise to another type of language bias, if studies with particular characteristics are more likely to be published in non‐English languages because, for instance, they are deemed unsuitable for international journals. For example, studies conducted on particular ecosystems, at particular intervention intensities, or conducted by local practitioners who do not speak English, could be systematically omitted from meta‐analyses (*language bias in study characteristics* in Figure 1). Considering that up to 36% of scientific studies on biodiversity conservation is published in languages other than English (Amano, González‐Varo, & Sutherland, 2016), and that non‐English studies are typically omitted from ecological meta‐analyses, an assessment of the impacts of language bias on ecological inferences drawn from meta‐analyses is urgently needed.

Here, we address this knowledge gap and assess the risk of language bias on outcomes of meta‐analyses. We searched for published peer‐reviewed meta‐analysis articles that analyzed sufficient numbers of both English‐ and Japanese‐language peer‐reviewed studies (10 or more effect‐size estimates in each language). The Japanese language was chosen not only for a practical reason (most of the authors are native Japanese‐language speakers) but also because the Japanese language is one of the major non‐English languages for scientific documentation in biodiversity conservation (Amano et al., 2016). We first tested the differences in effect‐size estimates between English‐ and Japanese‐language studies included in eligible meta‐analyses, to quantify the impacts of excluding the Japanese‐language studies on the overall mean effect sizes. To investigate the possible causes of differences in effect‐size estimates between the languages, we then tested for language bias in study characteristics by investigating between‐language differences in study characteristics that were deemed important in modifying the effects in the original meta‐analyses (i.e., potential effect modifiers). Next, we quantified differences in effect‐size estimates between the languages after controlling for the revealed differences in the study characteristics. Finally, we discuss the processes through which language bias may arise and propose guidelines for incorporating non‐English‐language studies in ecological meta‐analyses.

## 2 MATERIALS AND METHODS

### 2.1 Systematic literature search

We searched for ecological meta‐analysis articles including both English‐language and Japanese‐language studies. We performed searches in Web of Science Core Collection (https://webofknowledge.com/), CAB Direct (https://www.cabdirect.org/), and Wiley Online Library (https://onlinelibrary.wiley.com/) using English‐language search strings via Bangor University institutional access, and in CiNii (https://ci.nii.ac.jp/), which is the largest and most comprehensive database in Japan, using a Japanese‐language search string with no subscription (Appendix S1). We also performed Web‐based searches on Google Scholar (in English: https://scholar.google.com/; in Japanese: https://scholar.google.co.jp/) using an English‐language search string and a Japanese‐language search string (Appendix S1). Each search string contains meta‐analy* OR "meta analy*." We tailored a search string for each bibliographic platform, database, and Web‐based search engine (Appendix S1). We used translated version of the terms for the searches in Japanese (AppendixS1). All searches were conducted in Gwynedd, Wales, United Kingdom, on 19, 24, and 25 July 2018, and the searches were updated on 29 March 2019 (see exact time and date for each search in AppendixS1 https://refworks.proquest.com/); Mendeley (https://www.mendeley.com/); and EndNote Basic (https://endnote.com). Prior to the full searches and result retrieval, we had developed and predefined searches and result retrieval through a pilot test (AppendixS2 and AppendixS3). For the pilot searches, we adopted search strings developed by O’Leary et al. (2016).

### 2.2 Eligibility screening

Our search strings retrieved 1,504 unique articles. Duplicates were removed using Mendeley's “Check for Duplicates” tool and manually. We then screened articles according to titles and abstract, followed by full texts. We screened these articles to obtain meta‐analysis articles on ecological or evolutionary topics, suitable for assessing possible language biases. We included meta‐analysis articles that expressed the outcome of multiple studies on a common scale, through the calculation of an “effect size” for each study, which represents the magnitude of a difference between control and treatment means (e.g., log response ratio, standardized mean difference). We included meta‐analysis articles that aimed to quantitatively combine effect sizes to yield an overall estimate, or attribute variation in effect sizes to meaningful covariates using meta‐regression. Note that a meta‐analysis article can include multiple meta‐analyses. To identify meta‐analysis articles authored by individuals capable of searching literature in both English and Japanese, we included only meta‐analysis articles conducted by research teams with at least one author affiliated with a Japanese institution. Finally, the meta‐analyses had to include 10 or more effect‐size estimates published in both English and Japanese languages (AppendixS4). Articles meeting all of these eligibility criteria were included in our analysis. We used a modified version of the ROSES Flow Diagram for Systematic Review for reporting the number of articles retrieved at each screening stage (Haddaway, Macura, Whaley, & Pullin, 2017, 2018) (AppendixS5). We do not report a critical appraisal component of the diagram, because we did not conduct critical appraisal of the meta‐analysis articles.

### 2.3 Data selection

We extracted effect‐size estimates provided by four eligible meta‐analyses published in three meta‐analysis articles by Koshida and Katayama (2018), Osada et al. (2013), and Spake et al. (2019). Koshida and Katayama (2018) included 64 effect‐size estimates from 35 studies on the effects of rice‐field abandonment on biodiversity. We excluded six effect‐size estimates from four studies from non‐peer‐reviewed reports to distinguish the effect of language bias from publication bias. The meta‐analysis of Osada et al. (2013) compared the effect of light on leaf life span of four types of plants: deciduous herbaceous plants; evergreen herbaceous plants; deciduous woody plants; and evergreen woody plants. Only data of evergreen woody plants had sufficient numbers of both English‐ and Japanese‐language studies. Eight unpublished studies were also excluded. Data provided by Spake et al. (2019) included effect sizes representing forestry impacts on the species richness and abundance of three population groups: ground‐layer plants; saplings and seedlings; and invertebrates. Sufficient numbers of both English‐ and Japanese‐language studies were available for meta‐analyses of thinning impacts on the abundance of ground‐layer plants, and saplings and seedlings. Thus, we excluded data of the effect of thinning on species richness and on abundance of invertebrates.

As a result, we used 58 effect‐size estimates: 11 from six English‐language studies and 47 from 25 Japanese‐language studies from Koshida and Katayama (2018) (“rice‐field meta‐analysis” from hereon; Table 1 and Appendix S6). We also used 134 effect‐size estimates: 100 from 13 English‐language studies and 34 from two Japanese‐language studies from Osada et al. (2013) (“leaf life span meta‐analysis” from hereon; Table 1 and Appendix S7). From Spake et al. (2019), we used 65 effect‐size estimates: 41 from six English‐language studies and 24 from three Japanese‐language studies on the effect of thinning on abundance of ground‐layer plants (“plant forestry meta‐analysis” from hereon; Table 1 and Appendix S8), and 41 effect‐size estimates: 26 from six English‐language studies and 15 from four Japanese‐language studies on the effect of thinning on abundance of saplings and seedlings (“sapling forestry meta‐analysis” from hereon; Table 1 and Appendix S9).

**Table 1:** Details of eligible meta‐analyses, the number of studies, and the number of effect‐size estimates (total, in English, and in Japanese) included in each meta‐analysis

Meta‐analysis articles	Meta‐analyses	Number of studies			Number of effect‐size estimates		
		Total	English	Japanese	Total	English	Japanese
Koshida and Katayama (2018)	Rice‐field meta‐analysis	31	6 (19%)	25 (81%)	58	11 (19%)	47 (81%)
Osada et al. (2013)	Leaf life span meta‐analysis	15	13 (87%)	2 (13%)	134	100 (75%)	34 (25%)
Spake et al. (2019)	Plant forestry meta‐analysis	9	6 (67%)	3 (33%)	65	41 (63%)	24 (37%)
	Sapling forestry meta‐analysis	10	6 (60%)	4 (40%)	41	26 (63%)	15 (37%)
	Total	62	29 (47%)	33 (53%)	298	178 (60%)	120 (40%)

Note

The two meta‐analyses in Spake et al. (2019) used some studies in common, and thus, the total number of studies does not equate to the sum of studies used in each meta‐analysis. Meta‐analysis article: a published article (paper) that conducted at least one relevant meta‐analysis. Meta‐analysis: a statistical analysis of multiple effect‐size estimates measuring the effect of an intervention on a distinct group of subjects. Study: a paper included in a meta‐analysis, providing at least one effect‐size estimate. Effect‐size estimate: effect sizes estimated from data published in original primary studies and used in a meta‐analysis.

### 2.4 Data analysis

#### 2.4.1 Effect‐size difference between languages

**Figure 2:**
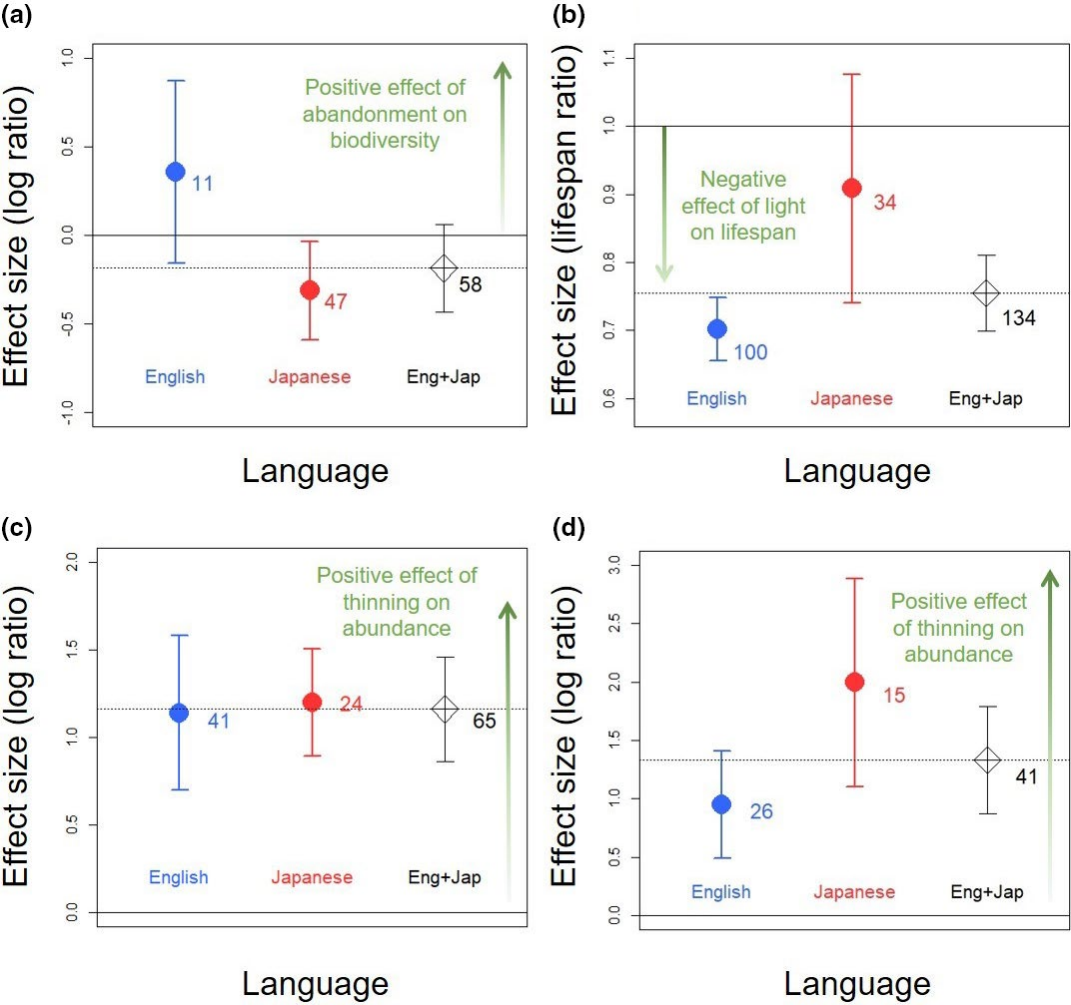
Differences in mean effect sizes between English‐ (blue) and Japanese‐language studies (red). (a) Koshida and Katayama (2018) showing the effects of rice‐field abandonment on biodiversity (rice‐field meta‐analysis). (b) Osada et al. (2013) showing the effect of light on plants’ leaf life span (leaf life span meta‐analysis). (c) Spake et al. (2019) showing the effect of thinning on ground‐layer plant abundance (plant forestry meta‐analysis). (d) Spake et al. (2019) showing the effect of thinning on sapling and seedling abundance (sapling forestry meta‐analysis). The number of effect‐size estimates in each language is also shown. The error bars show 95% confidence intervals. Diamonds and dotted lines show mean effect sizes estimated from all studies pooled (i.e., English‐ + Japanese‐language studies)

We first tested homogeneity of variance and normality of effect‐size estimates (log response ratio and life span ratio) using Levene's test and two‐sample Kolmogorov–Smirnov test, respectively. We then tested differences in effect‐size estimates between English‐language studies and Japanese‐language studies, using a two‐sample *t* test or Welch two‐sample *t* test (Ruxton, 2006) where the assumption of homogeneity of variance was not met. While these analyses assume independence between effect‐size estimates, some studies included in each meta‐analysis have multiple effect‐size estimates. Although some of those effect‐size estimates (e.g., those estimated at different locations) can be assumed as independent comparisons, others may not, for example, by sharing common control groups. We could not however include study as a random effect (a common solution in meta‐analyses, e.g., see Spake et al., 2019), because (a) an unbalanced number of effect‐size estimates in each study (ranging from 1 to 36 in our analyses) can lead to unstable parameter estimates (Harrison et al., 2018), and (b) language is a study‐level variable and can therefore be confounded with the random effect of each study. Therefore, our statistical tests may be vulnerable to increased type I errors. However, even if effect‐size estimates are not independent to each other, the estimation of a mean effect size is not affected by this (Borenstein et al., 2009), and thus, mean effect sizes in each language shown in Figures 2 and 6 should be reliable.

Meta‐analyses typically estimate precision‐weighted mean effect sizes, with each effect size weighted by the inverse of its variance (in addition to between‐study variance). Studies that omit the required information to estimate effect‐size variance may thus be excluded (Borenstein et al., 2009). Therefore, as a sensitivity analysis, we repeated the above analyses but on a subset of effect‐size estimates from studies that reported corresponding variance measures. We did not perform this sensitivity analysis for Osada et al. (2013) because the original meta‐analysis did not provide standard deviations of effect‐size estimates.

#### 2.4.2 Language bias in study characteristics

We tested associations between the languages and study characteristics using two‐way chi‐squared tests and Mann–Whitney *U* tests. For Koshida and Katayama (2018), taxa, management types, landscape types, soil types, and outcomes measured were analyzed. For Osada et al. (2013), measurement conditions, study countries, and plant families were analyzed. Study countries were tested because Japanese‐language studies are not necessarily conducted in Japan. In the case of Spake et al. (2019), intervention intensity and stand age were analyzed. We chose these variables because the original meta‐analyses treated the variables as potential effect modifiers and they were available for analyses. Note that although strictly speaking these are the characteristics of each effect‐size estimate, many of those characteristics are usually determined at the study level; hence, we used the term “study characteristics” instead of “characteristics of effect‐size estimates.” The statistical tests were conducted in R version 3.5.0 (R Core Team, 2018).

#### 2.4.3 Language bias in statistical results

We used two approaches to assess whether effect‐size estimates differed between languages after controlling differences in study characteristics. First, for each meta‐analysis, we fitted linear models with effect‐size estimates as the response variable, with explanatory variables including publication language in addition to factors that had a significant association with language (see above) as fixed factors, then compared two models: with and without the fixed factor language. Second, we fitted linear mixed models with effect‐size estimates as the response variable, publication language as the fixed factor, and factors that had a significant association with language as random factors, and tested the significance level of the fixed factors using likelihood‐ratio tests with the reduced models (Quinn & Keough, 2002). We ran the linear mixed models and performed likelihood‐ratio tests using lme4 package (Bates, Mächler, Bolker, & Walker, 2015) in R version 3.5.0 (R Core Team, 2018).

## 3 RESULTS

### 3.1 Searches and screening

Of the 1,504 unique articles retrieved by our search strings, 40 articles met our inclusion criteria as ecological meta‐analyses conducted by at least one author affiliated with a Japanese institution. These comprised meta‐analyses from a wide range of subdisciplines, including forestry, phenology, agriculture, and ecosystem services. Only three published meta‐analysis articles searched for studies published in both English and Japanese languages. Thirty‐six English‐language meta‐analysis articles included studies published only in English, while one Japanese‐language meta‐analysis article synthesized only Japanese‐language studies (listed as “Evidence base” for the reason for exclusion in AppendixS10). From the three articles meeting our criteria, four separate meta‐analyses had sufficient data to examine potential language bias effects (Table 1; also see Section 2.3 Data Selection in Methods). Japanese‐language studies constituted 81% of the effect‐size estimates from the rice‐field meta‐analysis, 25% from the leaf life span meta‐analysis, and 37% each from the two forestry meta‐analyses (Table 1).

### 3.2 Effect‐size differences between languages

Effect sizes representing the effect of rice‐field abandonment on biodiversity differed between English‐ and Japanese‐language studies from the rice‐field meta‐analysis (Table 2, Figure 2a). Both the magnitude and direction of the mean effect sizes differed among languages. A positive mean effect was estimated from English‐language‐only studies, whereas a negative mean effect was estimated from Japanese‐language‐only studies, and from all studies pooled (Figure 2a). This result did not change when only a subset of effect‐size estimates that had reported standard deviations were analyzed (i.e., those that allow weighted meta‐analysis: AppendixS11.

**Table 2:** Results of statistical tests for homogeneity of variance, normality, and differences in effect sizes between English‐ and Japanese‐language studies

Meta‐analysis	Levene's test for homogeneity of variance		Two‐sample Kolmogorov–Smirnov test for normality		Two‐sample *t* test for effect‐size differences between languages	
	*F* (*df*)	*p*	*D*	*p*	*t* (*df*)	*p*
Rice‐field meta‐analysis	0.13 (1, 56)	.72	0.44	.06	2.18 (56)	**.03**
Leaf life span meta‐analysis	4.55 (1, 132)	**.03**	0.27	.08	−2.40 (38.42)	**.02**
Plant forestry meta‐analysis	1.68 (1, 63)	.20	0.29	.12	−0.19 (63)	.85
Sapling forestry meta‐analysis	6.07 (1, 39)	**.02**	0.36	.17	−2.03 (21.62)	.05

Note

Statistically significant results are in bold. Welch two‐sample *t* test was used where the assumption of homogeneity of variance was not met.

Similarly, effect sizes for the leaf life span meta‐analysis differed between the languages (Table 2, Figure 2b). . The mean effect size estimated from English‐language‐only studies showed a more strongly negative effect of light on leaf life span than mean effect sizes estimated from both Japanese‐language studies and all studies, by 23% and 7%, respectively (Figure 2b).

In contrast, effect sizes did not differ significantly between English‐language and Japanese‐language studies from the plant forestry and sapling forestry meta‐analyses (Table 2). However, mean effect sizes from English‐language‐only studies in the sapling forestry meta‐analysis were 52% and 29% smaller than those estimated from Japanese‐language‐only studies and from all studies, respectively ( Figure 2d). Results differed for comparisons based on the subset of effect‐size estimates that were associated with standard deviations (i.e., those that allow weighted meta‐analysis). Effect sizes differed significantly between languages in the plant forestry meta‐analysis. The mean effect size estimated from Japanese‐language‐only studies was 318% more positive than the mean effect from English‐language‐only studies in the plant forestry meta‐analysis (*t* = −2.85; *df* = 29; *p* = .008; AppendixS12). The difference remained nonsignificant in the sapling forestry meta‐analysis (*t* = −0.98; *df* = 27; *p* = .33).

### 3.3 Language bias in study characteristics

**Figure 3:**
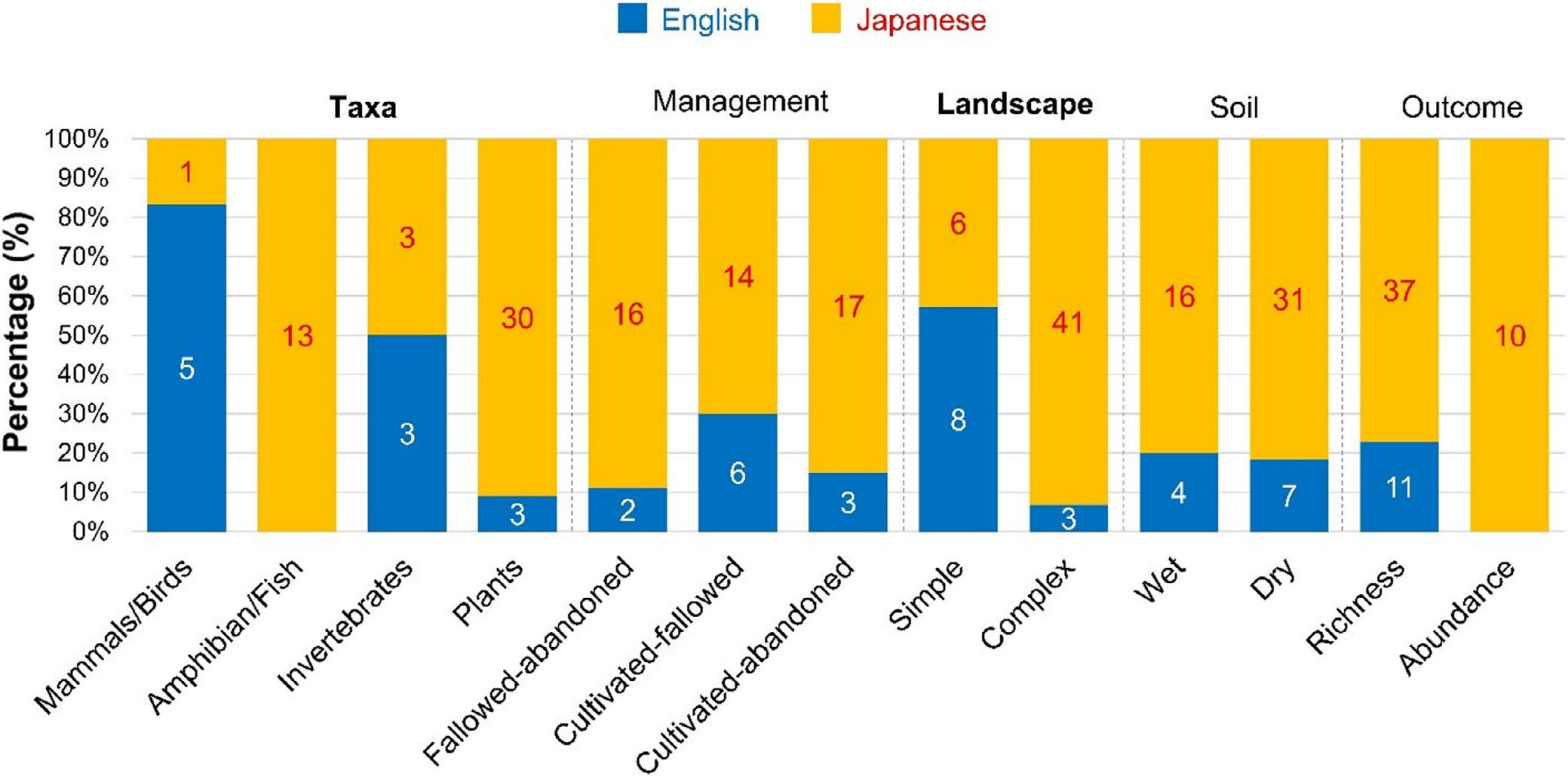
Differences in study characteristics between English‐ (blue) and Japanese‐language studies (orange) for the rice‐field meta‐analysis (Koshida & Katayama, 2018). Characteristics with a significant difference between the languages are in bold. The number of effect‐size estimates in each language is also shown in each bar

For the rice‐field meta‐analysis, although all of the included studies were conducted in Japan, the proportion of Japanese‐language studies varied significantly among taxa (*x* ^2^ = 25.07; *df* = 3; *p* < .001) and landscape types (*x* ^2^ = 14.38; *df* = 1; *p* < .001; Figure 3). Almost all studies on amphibians, fish, and plants and in complex landscapes were those published in Japanese (Figure 3).

**Figure 4:**
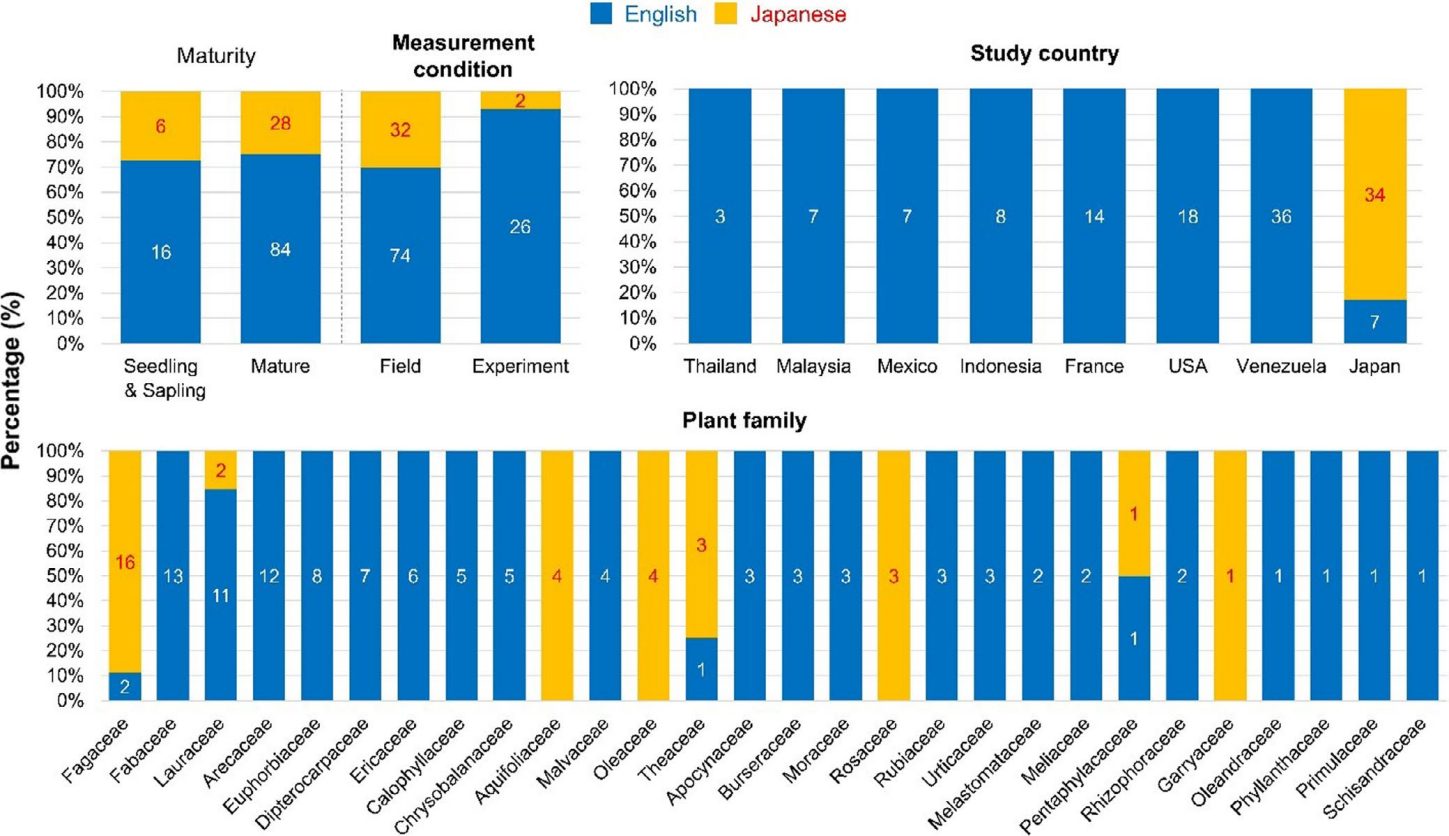
Differences in study characteristics between English‐ (blue) and Japanese‐language studies (orange) for the leaf life span meta‐analysis (Osada et al., 2013). Characteristics with a significant difference between the languages are in bold. The number of effect‐size estimates in each language is also shown in each bar

For the leaf life span meta‐analysis, the proportion of Japanese‐language studies differed significantly among measurement conditions (*x* ^2^ = 5.05; *df* = 1; *p* = .02), plant families (*x* ^2^ = 109.07; *df* = 27; *p* < .001), and not surprisingly study countries (*x* ^2^ = 103.34; *df* = 7; *p* < .001; Figure 4). Most studies with experimental measurements were those published in English, while all studies on four families, Aquifoliaceae, Oleaceae, Rosaceae, and Garryaceae, were those published in Japanese (Figure 4).

**Figure 5:**
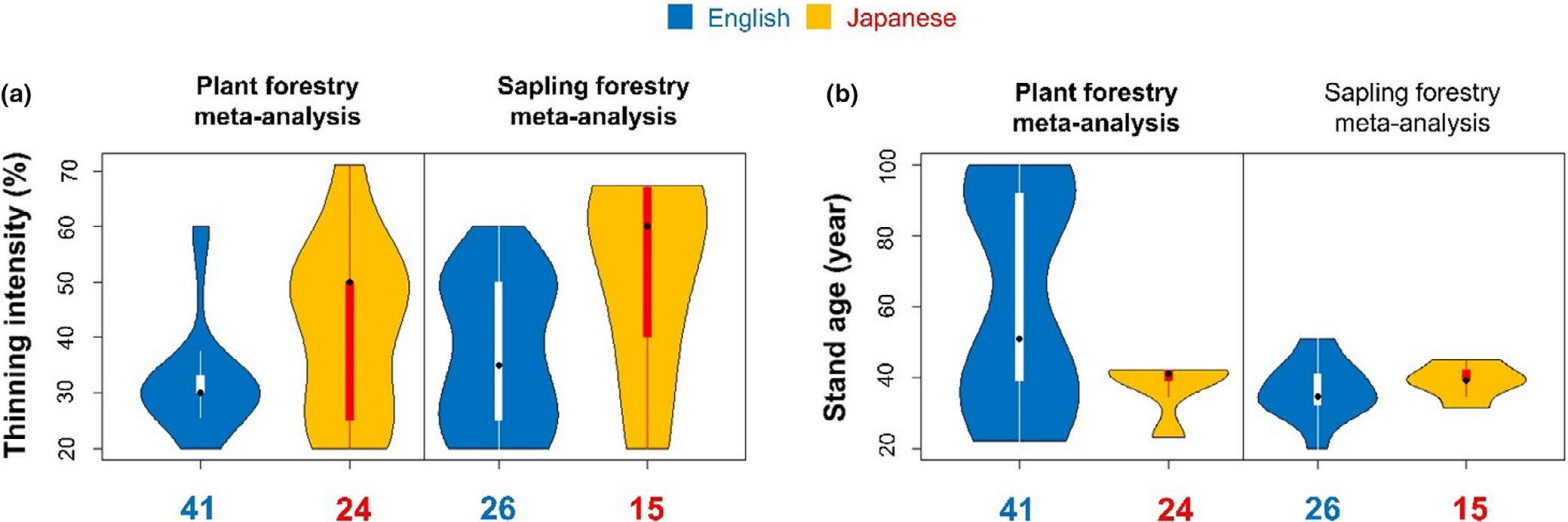
Differences in two study characteristics, (a) thinning intensity and (b) stand age, between English‐ (blue) and Japanese‐language studies (orange) for the plant and sapling forestry meta‐analyses (Spake et al., 2019). Black circles show medians, squares show interquartile ranges, and outer lines show ranges. The number of effect‐size estimates in each language is also shown below each panel

In the forestry meta‐analyses, the moderating influences of forest thinning intensity (volume removed, %) and stand age on effect sizes were analyzed (Spake et al., 2019). Despite the fact that all studies were conducted in Japan, thinning intensities were higher for studies published in Japanese than English for both the plant forestry meta‐analysis (*U_lower_* = 350; n1 = 41; n2 = 24; *p* = .049) and the sapling forestry meta‐analysis (*U_lower_* = 109; n1 = 26; n2 = 15; *p* = .02; Figure 5a). Stand age was younger in Japanese‐language studies for the plant forestry meta‐analysis (*U_lower_* = 646; n1 = 41; n2 = 24; *p* = .04), but it did not differ significantly between the languages for the sapling forestry meta‐analysis (*U_lower_* = 168; n1 = 26; n2 = 15; *p* = .47; Figure 5b). This difference remained when reanalyzed for the subset of effect‐size estimates associated with sample sizes and standard deviations for the plant forestry meta‐analysis (*U_lower_* = 54.5; n1 = 13; n2 = 18; *p* = .01); however, stand age did not differ significantly between the languages (*U_lower_* = 131; n1 = 13; n2 = 18; *p* = .58).

### 3.4 Language bias in statistical results

**Figure 6:**
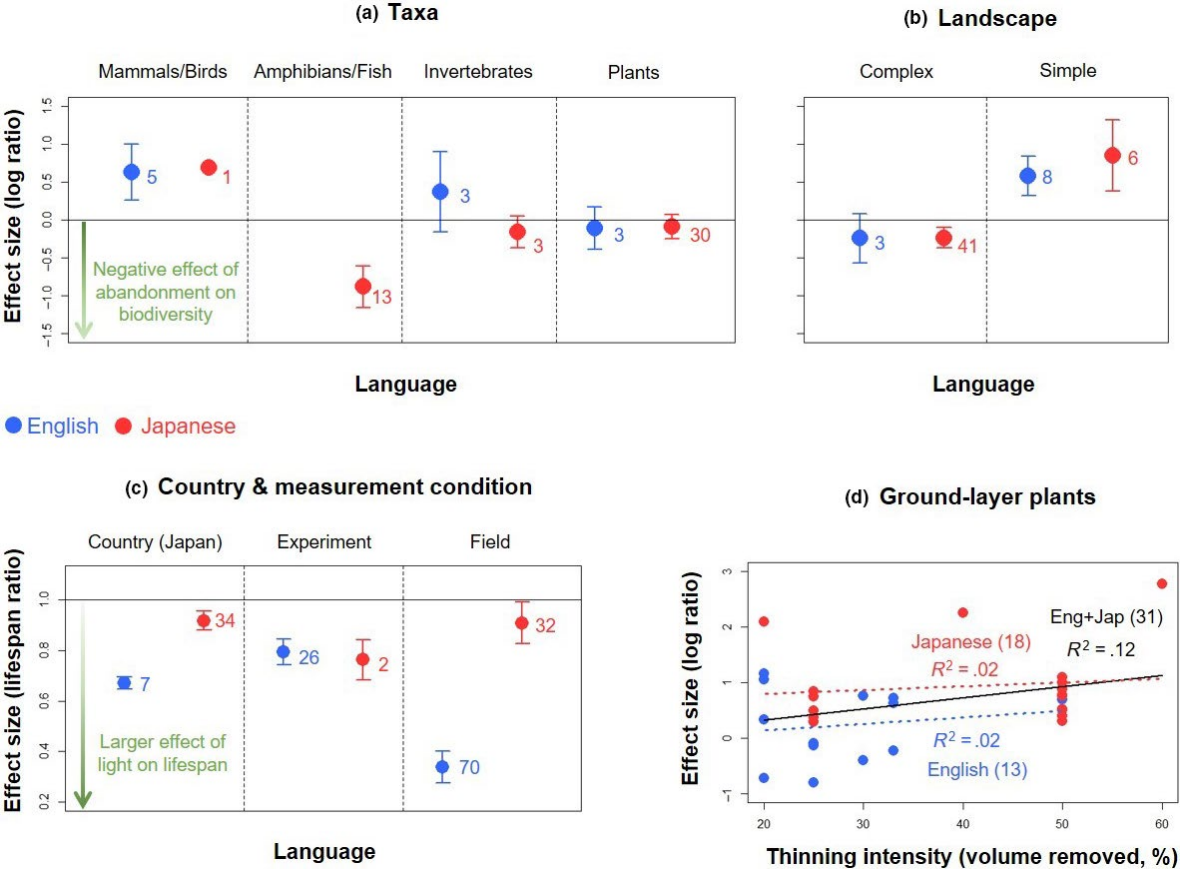
Tests for language bias in statistical results after controlling for the differences in study characteristics. Differences in effect sizes between English‐ (blue) and Japanese‐language studies (red) for (a) each taxon and (b) each landscape type in Koshida and Katayama (2018), for (c) studies conducted in Japan, and each measurement condition in Osada et al. (2013), and when (d) the effect of thinning intensity is controlled for in the plant forestry meta‐analysis (Spake et al., 2019). The number of effect‐size estimates in each language is also shown. The error bars show standard errors. In (d), black line shows the regression line based on all effect‐size estimates associated with standard deviations and sample sizes, while red and blue dotted lines show the regression lines based only on effect‐size estimates in English‐ and Japanese‐language studies reporting standard deviations and sample sizes, respectively

We next tested whether differences in effect sizes among languages remained after controlling for differences in study characteristics between the English‐language and Japanese‐language studies. In the rice‐field meta‐analysis, effect sizes did not differ significantly between the languages when the effects of taxa and landscape types were controlled for (Table 3, Figure 6a,b). Thus, the differences in effect sizes between the languages shown in Figure 2a seem to be largely attributable to the differences in study characteristics, together with the differences in effect sizes between taxa and landscape types (Figure 6a,b).

**Table 3:** Comparisons of models for testing effect‐size differences between languages, with and without language as a fixed factor

Meta‐analysis	Fixed factor(s)	Random factor(s)	Comparison of models with and without language as the fixed factor		
			*F*	*x* ^2^	*p*
Rice‐field meta‐analysis	Language + Taxa		0.26		.62
	Language + Landscape		0.14		.71
	Language	Taxa		1.97	.16
	Language	Landscape		0.58	.45
	Language	Taxa + Landscape		0.17	.68
Leaf life span meta‐analysis	Language + Measurement condition		12.64		**.0005**
	Language + Plant family		12.76		**.0005**
	Language	Measurement condition		10.68	**.001**
	Language	Plant family		13.59	**.0002**
	Language	Study country		16.00	**<.0001**
	Language	Measurement condition + Plant family + Study country		16.10	**<.0001**
Plant forestry meta‐analysis	Language + Thinning intensity		4.23		**.049**
	Language	Thinning intensity		12.06	**.0005**

Note

Statistically significant results (in bold) indicate that effect sizes differ between English‐ and Japanese‐language studies even after controlling for the relevant fixed or random factor(s).

In contrast, effect sizes from the leaf life span meta‐analysis still differed among languages after controlling for the effects of measurement conditions, plant families, and study countries (Table 3, Figure 6c). English‐language studies tended to report larger effect sizes than the Japanese‐language studies (Figure 6c), even for studies conducted only in Japan or those with field measurements, indicating a systematic difference in reported statistical results.

Effect‐size estimates associated with standard deviations from the plant forestry meta‐analysis differed among languages after controlling for the difference in forest thinning intensity (Table 3, Figure 6d). This again suggests a systematic difference in reported statistical results between languages, but in this case, the Japanese‐language studies tended to report larger effect sizes than the English‐language studies (Figure 6d).

## 4 DISCUSSION

Our reanalysis of four published meta‐analyses has revealed differences in mean effect sizes from studies published in English and Japanese. Specifically, two out of the four multiple‐language ecological meta‐analyses (and in another, when focusing only on effect‐size estimates associated with standard deviations) had effect sizes that differed by language. Although most of the ecological meta‐analyses identified in our initial screening neither searched for nor included studies published in multiple languages, Japanese‐language studies constituted a high proportion of effect‐size estimates included in the four eligible meta‐analyses (Table 1). These results suggest that English‐language studies do not necessarily comprise a random subset of the global literature, and thus, ignoring non‐English‐language studies in ecological meta‐analyses may lead to biased estimates of overall mean effect sizes, and biased inferences about ecological effects. This is a serious concern, given that meta‐analyses are often used to inform decision‐making in conservation policy and practice across a range of contexts (Gurevitch et al., 2018).

Our results also illustrate that language bias in study characteristics could lead to biased estimates of overall mean effect sizes, if non‐English‐language studies are ignored. This is a potential threat to ecological meta‐analyses, which typically synthesize heterogeneous studies on multiple species, conducted at multiple study scales (temporal, spatial) and with different methods (e.g., observational, experimental) (Spake & Doncaster, 2017). It is especially hard, or even impossible, to address the effect of language bias in study characteristics on overall mean effect sizes, when none or only a few effect‐size estimates are available in English for some study characteristics (e.g., effect‐size estimates on amphibians, fish, and plants in Koshida & Katayama, 2018). As a substantial amount of scientific literature on specific ecosystems and endemic species seems to be published in non‐English languages (Amano et al., 2016), omitting non‐English‐language studies might result in biased samples of study characteristics, potentially leading to invalid inferences. The general risk of language bias in study characteristics might thus be higher for ecology than for medical sciences.

We discuss four principal reasons that might give rise to language bias in study characteristics for Japan. First, there are well‐established national and local societies in Japan that publish their own journals, such as the Ecological Society of Japan (http://www.esj.ne.jp/esj/), Japanese Institute of Landscape Architecture (https://www.jila‐zouen.org/), Crop Science Society of Japan (http://www.cropscience.jp/), and Japanese Society of Environmental Entomology and Zoology (http://kandoukon.org/). The journals published by these societies tend to be associated with particular subdisciplines or taxonomic groups (see AppendixS13 for 40 major Japanese‐language peer‐reviewed journals relevant to ecology and biodiversity conservation). For example, the Ecological Society of Japan has long promoted plant ecology, with over half of the journal's papers published on plants (Yamamichi & Hiraiwa‐Hasegawa, 2012). Such influences might motivate Japanese authors, including local practitioners who are not familiar with English, to publish their research on specific taxonomic groups in the Japanese‐language journals of those societies to which they hold membership(s). Second, research questions or hypotheses that require local traditional knowledge may tend to be published in Japanese. In the rice‐field meta‐analysis, Japanese‐language studies provided a particularly high proportion of effect‐size estimates for plants (90%; *N* = 30) and amphibians/fish (100%; *N* = 13) (Figure 3). These are species groups that have been most strongly affected by rice‐field abandonment in the traditional rural landscapes of Japan (known as *Satoyama* in Japanese) (Kidera et al., 2018; Koshida & Katayama, 2018). International journals often demand “generality” or “transferability” of study findings, and may judge studies on local topics in Japan as too system‐specific. Therefore, studies on plants, amphibians, and fish might be more likely to have been submitted to, and published in, Japanese‐language journals. Third, conservation scientists and applied ecologists may choose to publish in Japanese‐language journals in order to target a Japanese audience of policymakers and practitioners, who may not read English‐language articles. Finally, available resources (time, human, financial) might influence the choice of language for publication. In the leaf life span meta‐analysis, most Japanese‐language studies measured leaf life span in the field, while most English‐language studies employed experimental designs (Figure 4). As measuring leaf life span in the fields is less laborious compared to conducting experiments, this might suggest that studies conducted by more highly funded research groups are more likely to afford English‐language proofing, and be published in English‐language journals. Those potential processes through which language bias in study characteristics arises will need to be fully investigated in the future.

We found that the differences in effect sizes between the languages remained after controlling for the influence of covariates investigated in the leaf life span and plant forestry meta‐analyses. In the leaf life span meta‐analysis, between‐language differences in effect sizes were apparent even for studies conducted only in Japan, or those from field experiments (Figure6c). This finding might be explained by a general tendency for authors reporting smaller effects in non‐English languages, as has been observed in medical sciences (Egger etal.,^1997^), due to preemption that their results are not strong or interesting enough to be accepted by international English‐language journals. Such a tendency is only possible given the availability of local‐language journals, where authors can submit their works for publication. Hence, the existence of non‐English journals could in effect mitigate publication bias by providing non‐English‐language speakers with opportunities to report statistically insignificant results, but only if these journals are included in meta‐analyses.

Interestingly, we found the opposite effect of language bias in statistical results in the plant forestry meta‐analysis; Japanese‐language studies had larger mean effect sizes compared to English‐language studies (Appendix S12). One possible explanation for this is the issue of pseudoreplication (Davies & Gray, 2015; Spake & Doncaster, 2017), as pseudoreplicated study designs were prevalent in the contributing studies from Japan. While this finding still indicates that ignoring non‐English‐language studies could bias the estimation of mean effect sizes in ecological meta‐analyses, it also suggests that we should not always assume biases toward a certain direction (i.e., providing larger effects) unless relevant non‐English‐language studies are identified and incorporated appropriately.

Our study has several important caveats. First, our findings are drawn from only four meta‐analyses that met our inclusion criteria. This may partly be due to our criterion for meta‐analysis articles to have been published by at least one author affiliated to a Japanese institution. Relaxing this criterion might have increased the sample size. However, even with this restriction, we screened over 1,500 papers and identified 40 potentially relevant meta‐analysis articles, of which only three actively searched and included studies published in the two languages. Our small sample size therefore reflects a current common practice of ignoring non‐English‐language studies in ecological meta‐analyses. Second, our findings might depend on how the authors of the meta‐analysis articles collected, collated, and appraised the studies for their meta‐analyses. However, it was not possible to investigate whether these decisions influenced our results. Third, within these meta‐analyses, we divided studies into two smaller groups according to language, which may have led to increased type II errors of not detecting significant differences (e.g., in Figure 2c,d). Fourth, we explored meta‐analyses synthesizing studies published only in English and Japanese, and therefore, biases from other languages remain to be explored. Finally, it is possible that other study characteristics (e.g., geographical difference within Japan), not considered by the original meta‐analyses, might have better explained differences among effect sizes between the languages.

Despite being based on a small sample size, our findings have a broad, yet simple, implication for meta‐analyses in ecology and conservation science. Future meta‐analyses—particularly those conducted at global extents or in regions where English is not widely spoken—should actively search for relevant non‐English‐language studies and, if appropriate, include them. Searches for non‐English‐language studies could be implemented by collaboration with native speakers of the relevant non‐English languages (Walpole, 2019) or with the aid of emerging technologies (e.g., litsearchr package in R translates search strings into multiple languages: https://elizagrames.github.io/litsearchr/). The flip side is also true; ignoring English‐language studies could also introduce language bias, and therefore, even national‐level decision‐making in non‐English‐speaking regions requires rigorous assessment of studies in English and in relevant non‐English languages. Currently, the use of non‐English‐language studies in science is not common practice (Baethge, 2013; Neimann Rasmussen & Montgomery, 2018) or even discouraged (Lazarev & Nazarovets, 2018). However, our findings highlight the importance of re‐evaluating the role of non‐English scientific knowledge in science and a potential risk of ignoring it in meta‐analyses. Understanding the generality of our findings across languages, and quantifying any bias incurred from ignoring non‐English‐language studies, requires further research on other non‐English languages (including Spanish, Portuguese, and Chinese; Amano et al., 2016), in which large volumes of scientific literature are published.

## CONFLICT OF INTEREST

None declared.

## AUTHOR CONTRIBUTION

**Ko Konno:** Conceptualization (equal); Data curation (lead); Formal analysis (lead); Investigation (equal); Methodology (equal); Visualization (lead); Writing‐original draft (lead). **Munemitsu Akasaka:** Conceptualization (supporting); Formal analysis (supporting); Funding acquisition (supporting); Investigation (equal); Methodology (supporting); Supervision (supporting); Writing‐review & editing (equal). **Chieko Koshida:** Investigation (equal); Resources (equal); Writing‐review & editing (supporting). **Naoki Katayama:** Investigation (equal); Resources (equal); Writing‐review & editing (supporting). **Noriyuki Osada:** Investigation (equal); Resources (equal); Writing‐review & editing (supporting). **Rebecca Spake:** Investigation (equal); Methodology (supporting); Resources (equal); Writing‐review & editing (equal). **Tatsuya Amano:** Conceptualization (equal); Funding acquisition (lead); Investigation (equal); Methodology (equal); Project administration (lead); Supervision (lead); Writing‐review & editing (equal).

## Supporting information

Table S1

Table S2

Table S3

Table S4

Table S5

Table S6

Table S7

Table S8

Table S9

Table S10

Table S11

Table S12

Table S13

## Data Availability

Data are available in Supporting information.
