# Supplementary material for: Ignoring non‐English‐language studies may bias ecological meta‐analyses
Source: Ecol Evol. 2020 May 29;10(13):6373–84. doi: 10.1002/ece3.6368 (PMC7381574; doi:10.1002/ece3.6368)
Supplement: Supplementary file 3 — Table S3 [file ECE3-10-6373-s003.docx]

| Pre-defined method | We will use:   - RefWorks (<https://refworks.proquest.com/>) - Mendeley (<https://www.mendeley.com/>) - Zotero (<https://www.zotero.org/>) | |
| --- | --- | --- |
|  | Web of Science | Web of Science allows us to export retrieved articles as BIB file (.bib). BIB files will be imported into Mendeley. |
|  | CAB Direct | CAB Direct allows us to export retrieved articles into RefWorks. RIS file will be exported from RefWorks for importing into Mendeley. |
|  | Wiley Online Library | We will use Mendeley Web Importer (Mendeley’s internet search extension tool; <https://www.mendeley.com/reference-management/web-importer#id_1>) for Wiley Online Library. |
|  | CiNii | CiNii allows us to export retrieved articles as Text Document (.txt). We will import the files into RefWorks to convert them into RIS file. Then the RIS file will be imported into Mendeley. |
|  | Google Scholar | Google Scholar allows us to export retrieved articles into RefWorks. RIS file will be exported from RefWorks for importing into Mendeley. |
|  | Spreadsheet generation | Zotero will be used as an Excel file (.xlsx) generator. Once all the screening was done, the eligible articles and screened articles at full text level will be imported into Zotero to generate two spreadsheets: (i) a list of excluded articles at full text level; and (ii) a list of eligible articles. |
| Actual run | We used:   - RefWorks (<https://refworks.proquest.com/>) - Mendeley (<https://www.mendeley.com/>) - EndNote Online (<https://endnote.com>) | |
|  | Web of Science | No changes to pre-defined method. |
|  | CAB Direct | No changes to pre-defined method. |
|  | Wiley Online Library | No changes to pre-defined method. |
|  | CiNii | No changes to pre-defined method. |
|  | Google Scholar | We used Mendeley Web Importer for the search in English.  We were blocked (i.e. we could not import into Mendeley) by Google when searching in Japanese. We decided to print the 100 hits (as PDF), and then screened the search results. |
|  | Spreadsheet generation | We did not use Zotero. Instead, we used EndNote Online for generating a spreadsheet. Because we found a lot of blank cells in the Zotero-generated spreadsheet. |

**S3**. Pre-defined method for and actual run of search results retrieval.
