# Supplementary material for: Ignoring non‐English‐language studies may bias ecological meta‐analyses
Source: Ecol Evol. 2020 May 29;10(13):6373–84. doi: 10.1002/ece3.6368 (PMC7381574; doi:10.1002/ece3.6368)
Supplement: Supplementary file 4 — Table S4 [file ECE3-10-6373-s004.docx]

**S4**. Full eligibility criteria used at the screening step.

| **Population** | Any population of relevance to ecology, evolutionary biology or conservation science. |
| --- | --- |
| **Intervention/Exposure** | Intervention - Any intervention claimed to potentially achieve ecological or biodiversity benefits, or mitigation of ecological or biodiversity impacts.  OR  Exposure - Any exposure of relevance to biological evolution, or ecological or biodiversity impact. |
| **Comparator** | Any comparator which allows assessment of effect. |
| **Outcome** | Measure of effect of interest. |
| **Study type** | Review article using effect size to synthesise the outcome measure (i.e. meta-analysis) or examining heterogeneity of outcome measure (e.g. meta-regression).  We will exclude review article conducting primary research (e.g. experiment; i.e. mixture of review and primary research) and will also exclude Bayesian meta-analysis. |
| **Effect size** | The following effect sizes are eligible:   - Raw mean difference - Standardised mean difference - Ratio - Odds ratio - Log response ratio - Risk ratio - Risk difference - Fisher’s *z* transformed *r* |
| **Evidence base** | The review article must have included studies written in English and Japanese. The number of effect-size estimates in each language must be 10 or more for enabling meaning comparison of effect sizes between the languages. |
| **Subject scope** | Topic must have relevance to ecology, evolutionary biology or conservation science. A review having partial interests in this scope will be excluded. In other words, a review must have specific interests in these subjects. If clear link to the scope is NOT made, we will specifically exclude the following subjects:   - Veterinary science - Engineering and construction - Biotechnology and bioengineering - Human health care and medicine - Education - Social welfare and social justice - Toxicology - International development - Economics - Food science - Psychology |
| **Affiliation** | At least one of authors of a review must be affiliated with Japanese institution. This is based on the assumption that the authors have opportunity to include Japanese language studies in their review. |
