# Supplementary material for: Ignoring non‐English‐language studies may bias ecological meta‐analyses
Source: Ecol Evol. 2020 May 29;10(13):6373–84. doi: 10.1002/ece3.6368 (PMC7381574; doi:10.1002/ece3.6368)
Supplement: Supplementary file 5 — Table S5 [file ECE3-10-6373-s005.docx]

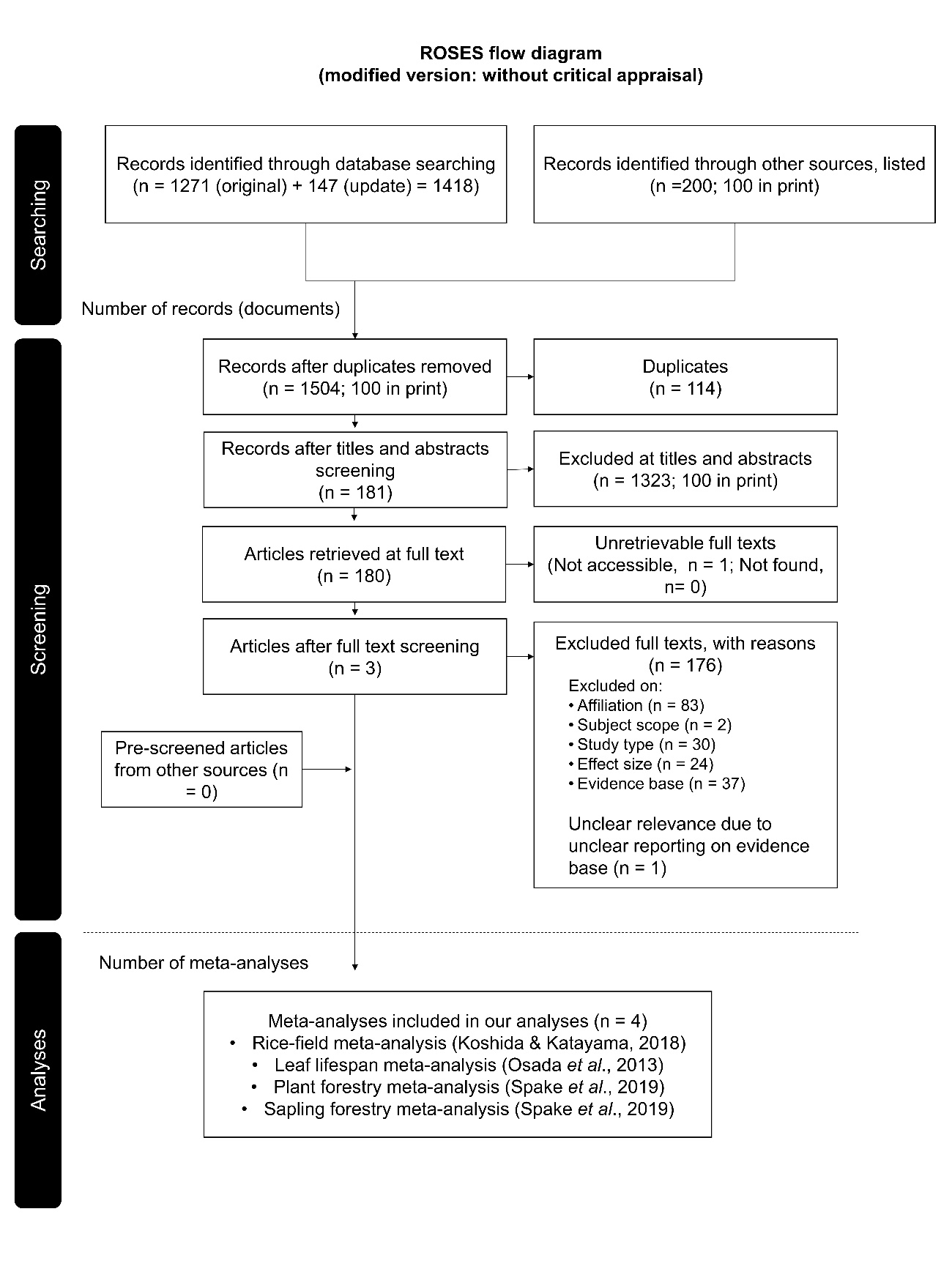


**S5**. Modified version of ROSES flow diagram. No critical appraisal was conducted during the process because critical appraisal should be conducted by independent appraisers. Note that we do not claim that our paper is a Systematic Review.
