# Supplementary material for: Ignoring non‐English‐language studies may bias ecological meta‐analyses
Source: Ecol Evol. 2020 May 29;10(13):6373–84. doi: 10.1002/ece3.6368 (PMC7381574; doi:10.1002/ece3.6368)
Supplement: Supplementary file 11 — Table S11 [file ECE3-10-6373-s011.docx]

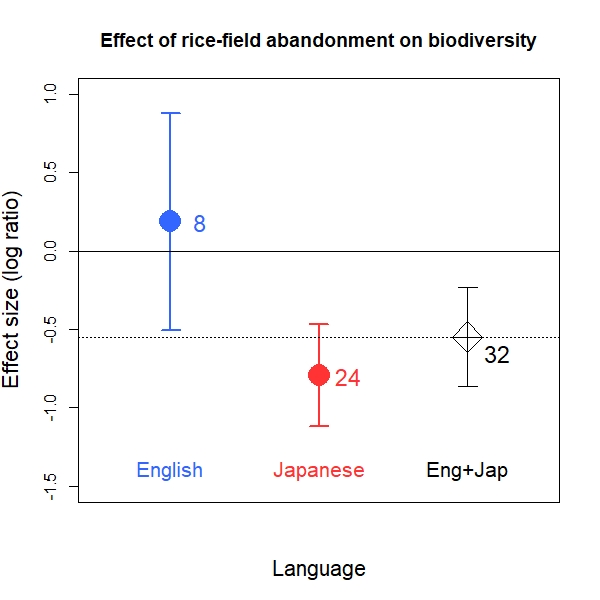


**S11**. Differences in effect size between languages, only with 32 (eight English and 24 Japanese) effect-size estimates associated with standard deviations (i.e., those that allow weighted meta-analysis) in the rice-field meta-analysis (Koshida & Katayama 2018). The arrows show 95% confidence intervals. Effect sizes differ significantly between English- and Japanese-language studies (t = 3.08; df = 30; p = 0.004).
