# Supplementary material for: Ignoring non‐English‐language studies may bias ecological meta‐analyses
Source: Ecol Evol. 2020 May 29;10(13):6373–84. doi: 10.1002/ece3.6368 (PMC7381574; doi:10.1002/ece3.6368)
Supplement: Supplementary file 12 — Table S12 [file ECE3-10-6373-s012.docx]

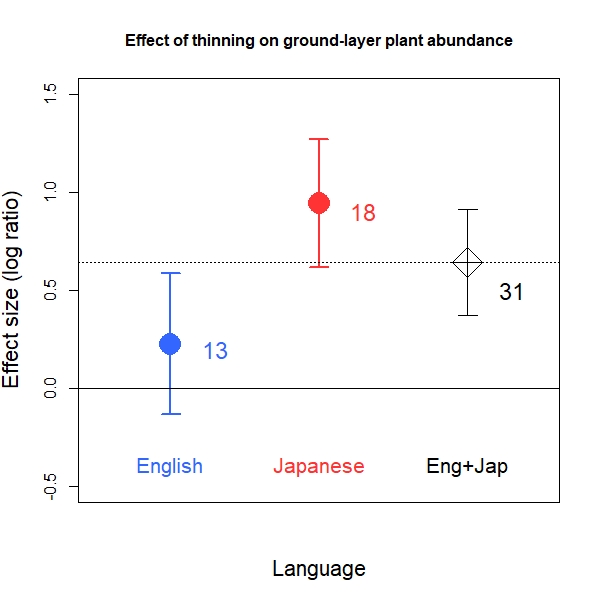


**S12**. Difference in effect size between languages, only with 31 (13 English and 18 Japanese) effect-size estimates associated with standard deviations (i.e., those that allow weighted meta-analysis) in the plant forestry meta-analysis (Spake et al. 2019). The arrows show 95% confidence intervals. Effect sizes differ significantly between English- and Japanese-language studies (*t* = -2.85; df = 29; *p* = 0.008).
